# Genetic basis for glandular trichome formation in cotton

**DOI:** 10.1038/ncomms10456

**Published:** 2016-01-22

**Authors:** Dan Ma, Yan Hu, Changqing Yang, Bingliang Liu, Lei Fang, Qun Wan, Wenhua Liang, Gaofu Mei, Lingjian Wang, Haiping Wang, Linyun Ding, Chenguang Dong, Mengqiao Pan, Jiedan Chen, Sen Wang, Shuqi Chen, Caiping Cai, Xiefei Zhu, Xueying Guan, Baoliang Zhou, Shuijin Zhu, Jiawei Wang, Wangzhen Guo, Xiaoya Chen, Tianzhen Zhang

**Affiliations:** 1 State Key Laboratory of Crop Genetics and Germplasm Enhancement, Cotton Hybrid R & D Engineering Center (the Ministry of Education), Nanjing Agricultural University, Nanjing 210095, China; 2 National Key Laboratory of Plant Molecular Genetics, National Plant Gene Research Center, Institute of Plant Physiology and Ecology, Shanghai Institutes for Biological Sciences, Chinese Academy of Sciences, Shanghai 200032, China; 3 Department of Agronomy, College of Agriculture and Biotechnology, Zhejiang University, Zhejiang 310029, China

## Abstract

Trichomes originate from epidermal cells and can be classified as either glandular or non-glandular. *Gossypium* species are characterized by the presence of small and darkly pigmented lysigenous glands that contain large amounts of gossypol. Here, using a dominant glandless mutant, we characterize *GoPGF*, which encodes a basic helix-loop-helix domain-containing transcription factor, that we propose is a positive regulator of gland formation. Silencing *GoPGF* leads to a completely glandless phenotype. A single nucleotide insertion in *GoPGF*, introducing a premature stop codon is found in the duplicate recessive glandless mutant (gl_2_gl_3_). The characterization of *GoPGF* helps to unravel the regulatory network of glandular structure biogenesis, and has implications for understanding the production of secondary metabolites in glands. It also provides a potential molecular basis to generate glandless seed and glanded cotton to not only supply fibre and oil but also provide a source of protein for human consumption.

## Abstract (web summary)

Cotton plants are characterized by the presence of darkly pigmented glands that accumulate metabolites that contribute to plant defence but limit the use of cottonseed as food. Here, Ma *et al*. provide evidence that GoPGF, a bHLH transcription factor, is responsible for gland formation.

## Graphical abstract

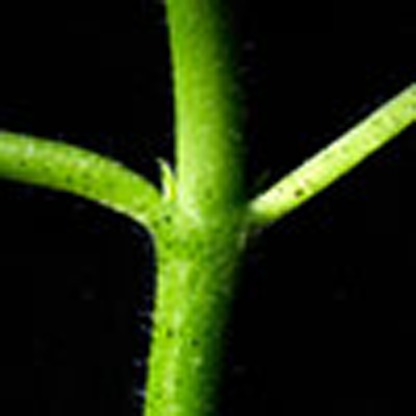

Trichomes are specialized structures that originate from epidermal cells, and can be classified into two main categories: non-glandular or glandular^1^. The most remarkable feature of glandular trichomes is their unique capacity to synthesize, store and sometimes secrete a wide array of metabolites, including polysaccharides, organic acids, proteins, terpenoids (such as the antimalarial drug artemisinin), alkaloids and polyphenols^1^,^2^. These compounds give plants a distinctive smell, the natural barrier to protect plants against herbivorous insects and pathogens, or have a significant commercial value as drugs, fragrances, food additives and insecticides^1^,^3^. As such, glandular trichomes have been described as bio-factories for the production of high-value natural products such as volatile oils, resins, mucilages and gums. Despite extensive study of the development and fine structure, and physiological and molecular metabolisms with regard to chemicals synthesized, stored and secreted in glandular trichomes^4^,^5^,^6^,^7^, almost nothing is known about the genetics underlying their development.

Cotton (*Gossypium*. spp) is not only the leading natural fibre resource, but also the third largest field crop in terms of edible oilseed tonnage. In addition to 21% oil, cottonseed has a relatively high protein content (23%). For every kilogram of fibre produced, 1.65 kg of seed is also collected. According to this ratio, the global cottonseed production could potentially provide protein for half a billion people annually^8^. However, the *Gossypium* species are characterized by the presence of small darkly pigmented lysigenous glands containing gossypol deposits, which are toxic to humans and monogastric animals. Therefore, cottonseed cannot be used to produce edible proteins or oils directly. Gossypol is a yellowish phenolic compound that contributes to the defence of cotton against pests, diseases and abiotic stresses^9^,^10^. Therefore, potentially the best approach would be to develop cotton that produces gossypol-free seed for human consumption and normal gossypol content in other tissues for protection from pests and pathogens by seed-specific genetic engineering of gland formation or gossypol biosynthesis. Ultra-low gossypol cottonseed lines have been developed using RNAi knockdown of δ-cadinene synthase gene(s) during seed development in *Gossypium hirsutum*^8^. The ultra-low gossypol trait is stable under field conditions and the foliage/floral organs contain wild-type levels of gossypol and related terpenoids, indicates it could be possible to enhance and expand the nutritional utility of the annual cottonseed output to fulfil the ever-increasing needs of humanity after the evaluation of its safety and nutritional efficacy^9^.

Understanding the molecular genetic basis of gossypol gland formation could provide additional methods to develop gossypol-free cotton seeds that could be produced efficiently and be adapted widely. The glands originate from a cluster of gland primodium cells, which differ from other cells in that they have a high-density cytoplasm and large nucleolus. The internal cells are then degraded and form a cavity known as a gland in the ground meristem^11^. From a cross between a cultivated and a primitive race of *G. hirsutum*, McMichael^12^,^13^ recovered a recessive mutant that eliminates all glands on the aerial plant parts and seeds (Fig. 1). This offered the possibility of cultivating glandless cotton, which could potentially help to alleviate hunger and protein shortages^14^. Up to now, genetic research has revealed that cotton gland formation is determined by a combination of at least six independent loci, *gl*_*1*_, *gl*_*2*_, *gl*_*3*_, *gl*_*4*_, *gl*_*5*_ and *gl*_*6*_ (ref. 15). The glandless phenotype is controlled by two pairs of duplicate recessive genes (*gl*_*2*_*gl*_*3*_) on chromosome (chr.) A12 and D12, respectively^15^,^16^. In addition, a single dominant glandless mutation in *G. barbadense* was discovered in Egypt following the irradiation of Giza 45 seeds with ^32^P, which results in glandless plants and seeds (Figs 1 and 2a). Genetic analysis revealed that it is a dominant allele at the *Gl*_*2*_ locus that is epistatic to *Gl*_*3*_, and the gene symbol *Gl*_*2*_^*e*^ was proposed^17^,^18^. A new strain of cotton that is homozygous for this new gene was released as Bahtim 110 in Egypt^19^ and Hai-1 in China^18^. These two mutants have been used to develop many glandless cultivars of both *G. hirsutum* and *G. barbadense* to produce little or no gossypol in seeds. For example, more than 20 glandless cultivars have been developed in China^20^. However, the molecular genetic basis for the formation of pigment glandular trichomes, which are storage organs of gossypol in cotton, remains unknown. Here, through a map-based cloning approach, we identify *Gossypium PIGMENT GLAND FORMATION GENE (GoPGF*) as the likely causative gene for the phenotype of glandless mutant cotton. This helps to uncover the molecular basis for formation of glandular trichomes and secondary substances such as gossypol in cotton.

## Results

### Map-based cloning of the dominant glandless gene *Gl*_*2*_^*e*^

We previously anchored the dominant glandless gene *Gl*_*2*_^*e*^ between two microsatellites (SSRs), NAU3778 and NAU2251b, with genetic distances of 9.27 and 0.96 cm, respectively in chr. 12A (ref. 21). On the basis of the genome sequence of *G. raimondii*^22^, *Gl*_*2*_^*e*^ was delimited within a 1-Mb interval on scaffold D8. We mined and developed 316 SSRs to screen the polymorphism between Hai-1 and TM-1 within this region (Supplementary Data 1). We used an enlarged mapping population including 2,197 individuals (Supplementary Table 1) and seven polymorphic SSRs, narrowed the *Gl*_*2*_^*e*^ locus to a 43-kb region flanked between w7954 and w5383 with genetic distances of 0.5 and 0.6 cM, respectively (Fig. 2b; Supplementary Data 1). Within this region, seven putative open reading frames (ORFs; Supplementary Table 2) are predicted based on our high-quality reference genome sequence of tetraploid cotton *G. hirsutum* acc. TM-1 (ref. 23). Quantitative RT–PCR (qPCR; Fig. 2c; Supplementary Fig. 1) revealed that only the expression level of ORF2 was altered, and was significantly lower in the dominant glandless mutant Hai-1 and the (Hai-1 × TM-1)F_1_ than in the glanded *G. hirsutum* acc. TM-1 and *G. barbadense* cv. Hai7124. Therefore, we considered ORF2 as a candidate gene for *Gl*_*2*_^*e*^.

We then isolated the full length genomic DNA of ORF2 from the glandless Hai-1 and its wild type, Giza 45, and found that the coding region is 1,428 bp in length with no intron and contains a predicted gene of unknown function. The protein is predicted to contain a conserved motif shared by bHLH and R2R3-MYB in the N-terminal motif (Pfam14215) from amino acid 20 to 202 and a helix-loop-helix DNA-binding domain (Pfam00010) from amino acid 293 to 350 at the C-terminal (http://pfam.sanger.ac.uk/), which is involved in DNA binding and protein oligomerization. The gene is phylogenetically closely related to bHLH transcription factor (Thecc1EG015640t1) in *Theobroma cacao* and belongs to bHLH transcription factor family. We named the gene as *Gossypium* pigment gland formation gene (*GoPGF*). Orthologous genes of *GoPGF* in *G. raimondii* and *G. arboreum* are Gorai.008G259000.1 and Cotton_A_01306, respectively. GoPGF protein was localized to the nucleus (Supplementary Fig. 2). Quantitative reverse transcriptase PCR (qRT–PCR) analysis revealed that the gene is expressed in a constitutive manner in most organs and tissues, including root, stem and leaf (Fig. 2c; Supplementary Fig. 3), consistent with the presence of glands. DNA sequence comparisons revealed that three single nucleotide polymorphisms (SNPs) exist between the dominant glandless Hai-1 and the glanded Giza 45 and other *G. barbadense* cultivars in *GbPGF* (Supplementary Fig. 4), but only result in one amino acid change from alanine to valine at residue 43 (Fig. 2d). On the basis of these SNPs, we found that the mutant allele (*GbPGFm*) from Hai-1 co-segregates with 1,624 glandless plants of three F_2_s (Supplementary Data 1).

The difference in *GoPGF* mRNA levels between Hai-1 and TM-1 may possibly be determined by transcriptional regulation of the *GoPGF* gene. To investigate the differential expression of *GoPGF_A12* from TM-1 and Hai-1, we isolated 1.8-kb fragments upstream of the start codon of *GoPGF* from TM-1 and Hai-1. No difference in transient expression of GUS reporter constructs was detected after *Agrobacterium*-mediated transformation of tobacco leaves (Supplementary Fig. 5), indicating that their promoters likely do not confer the extra low expression of *PGF* in Hai-1. Whether the mutation reduces its expression in Bahtim 110 or Hai-1 or whether a causative mutation on a distant loci^24^ exerts influence on *GbPGF* remains to be explored.

### Nucleotide insertion results in two recessive mutant alleles

**Figure 1:**
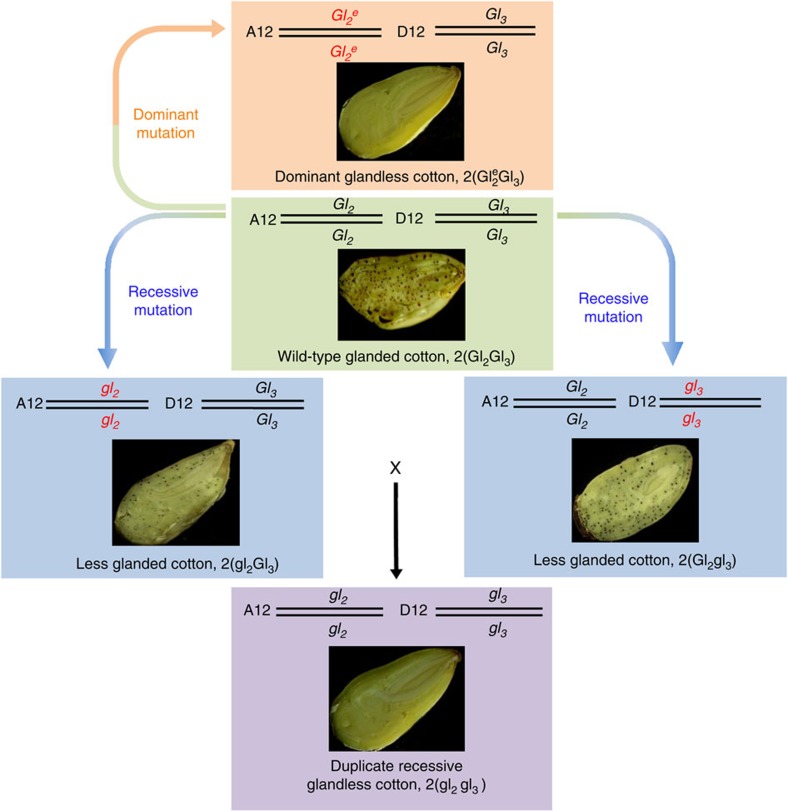
Origins of the genotypes and phenotypes of glandless tetraploid cottons. The genotypes include a dominant mutation, *Gl*_*2*_^*e*^*Gl*_*3*_; two recessive mutations, *gl*_*2*_*Gl*_*3*_ and *Gl*_*2*_*gl*_*3*_; and a duplicated recessive cotton, *gl*_*2*_*gl*_*3*_. Black points in each graph show the glands on the surface of seeds.

**Figure 2:**
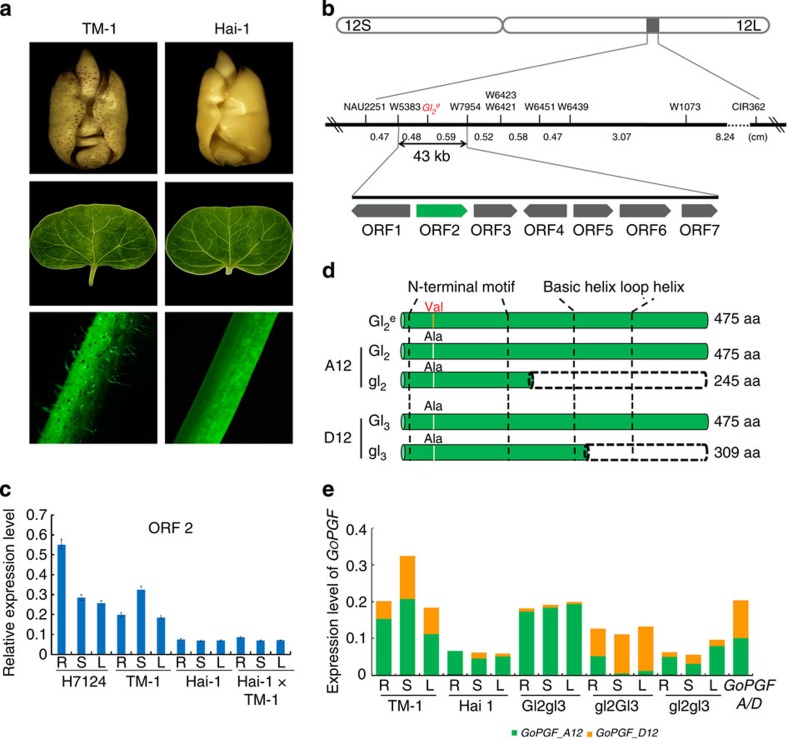
Fine mapping of dominant glandless gene *Gl* and the expression pattern of *GoPGF*. (**a**) Gland traits of seeds, leaves and stems of TM-1 (glanded) and Hai-1 (glandless). (**b**) Fine mapping of the dominant glandless gene, *Gl*. Seven genes (ORF1–7) in the mapping region are indicated by boxes. (**c**) Real-time RT–PCR expression analysis of ORF2 in the root, stem and leaf of Hai7124, TM-1, Hai-1 and Hai-1 × TM-1. Error bars represent the s.d. of the mean values of three biological replicates. (**d**) Sequence diversity of PGF. An amino acid change from alanine to valine was observed in the dominant glandless gene (Gl). The premature translation termination resulted in the production of fewer glands (Gl_2_gl_3_ and gl_2_Gl_3_ mutant) or completely glandless (gl_2_gl_3_ mutant) phenotypes. (**e**) Pyrosequencing analysis of the relative expression levels of *GoPGF_A12* and *GoPGF_D12* homoeologous alleles in the root, stem and leaf of TM-1, Hai-1, 2(Gl_2_gl_3_), 2(gl_2_Gl_3_) and 2(gl_2_gl_3_).

The whole plant glandless phenotype is controlled by a combination of two recessive mutant alleles, *gl*_*2*_ and *gl*_*3*_ (ref. 13) or one dominant gene *Gl*_*2*_^*e*^ (refs 13, 15; Fig. 1). So, if *PGF* is the candidate gene for *Gl*_*2*_^*e*^, we reasoned that *PGF_A12* and *PGF_D12* in the two recessive mutant alleles, *gl*_*2*_ and *gl*_*3*_, should be non-functional. We isolated and sequenced *GoPGFs* from tetraploid cotton accessions and their extant progenitor species, *G. arboreum* (A_2_) and *G. raimondii* (D_5_). Sequence alignments show that there are homoeologous *PGF* gene pairs in the corresponding A subgenome (*GoPGF_A12*) and D subgenome (*GoPGF_D12*) of tetraploid cottons with seven SNPs between them (Supplementary Fig. 4; Supplementary Table 3). We isolated the mutant *GoPGF* genes in the chr. A12 (*GhPGF_A12*^*m*^) and D12 (*GhPGF_D12*^*m*^), respectively, from the duplicate recessive glandless mutant, 2(gl_2_gl_3_), and the monomeric mutants 2(Gl_2_gl_3_) and 2(gl_2_Gl_3_) with a low number of glands (Fig. 1). Sequence alignments show that a single “T” nucleotide insertion occurs between 735 and 736 bp in the coding region of *GhPGF_A12*^*m*^ (*gl*_*2*_) in chr. A12, and a single “A” nucleotide insertion occurs between 916 and 917 bp in the coding region of *GhPGF_D12*^*m*^ (*gl*_*3*_) in chr.D12. This premature translation termination correlated with the production of fewer glands in monomeric mutants (Gl_2_gl_3_ and gl_2_Gl_3_) or completely glandless phenotype in the duplicate mutant (gl_2_gl_3_; Figs 1 and 2d; Supplementary Fig. 4). qPCR analysis revealed that *GhPGF_A12*^*m*^ (*gl*_*2*_) and *GhPGF_D12*^*m*^ (*gl*_*3*_) were expressed at very low levels compared to their corresponding homoeologous genes (*Gl*_*3*_ and *Gl*_*2*_) in the monomeric mutants 2(gl_2_Gl_3_) and 2(Gl_2_gl_3_), respectively, as well as in the duplicate recessive glandless mutant 2(gl_2_gl_3_) and in Hai-1 (Fig. 2e). These results strongly suggest that *GoPGF* is the gene that controls cotton pigment gland formation.

### *GbPGF*-silencing results in a glandless phenotype

**Figure 3:**
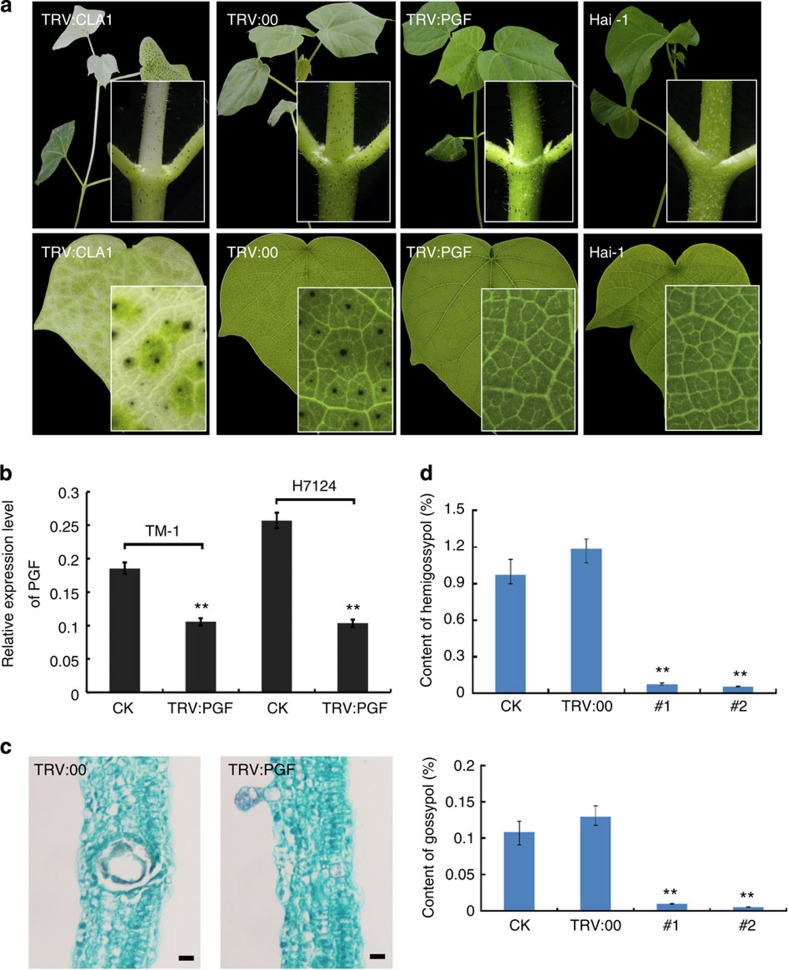
Functional characterization of *GoPGF* by VIGS. (**a**) Phenotypes of TM-1 before and after *GoPGF* silencing by VIGS, showing the presence and the absence of the glands. (**b**) Transcript level of *GoPGF* in *GoPGF*-silenced leaves. (**c**) Cavity observed in the leaves of TM-1 but disappeared in the leaves emerged after VIGS. Scale bar, 10 μm. (**d**) Hemigossypol and gossypol content in control (CK), empty vector (TRV:00) and in the *GoPGF*-silenced (#1 and #2) leaves of TM-1, determined by high-performance liquid chromatography. **P<0.01; Student's *t*-test, *n*=3. Error bars are s.d. of three biological repeats.

**Figure 4:**
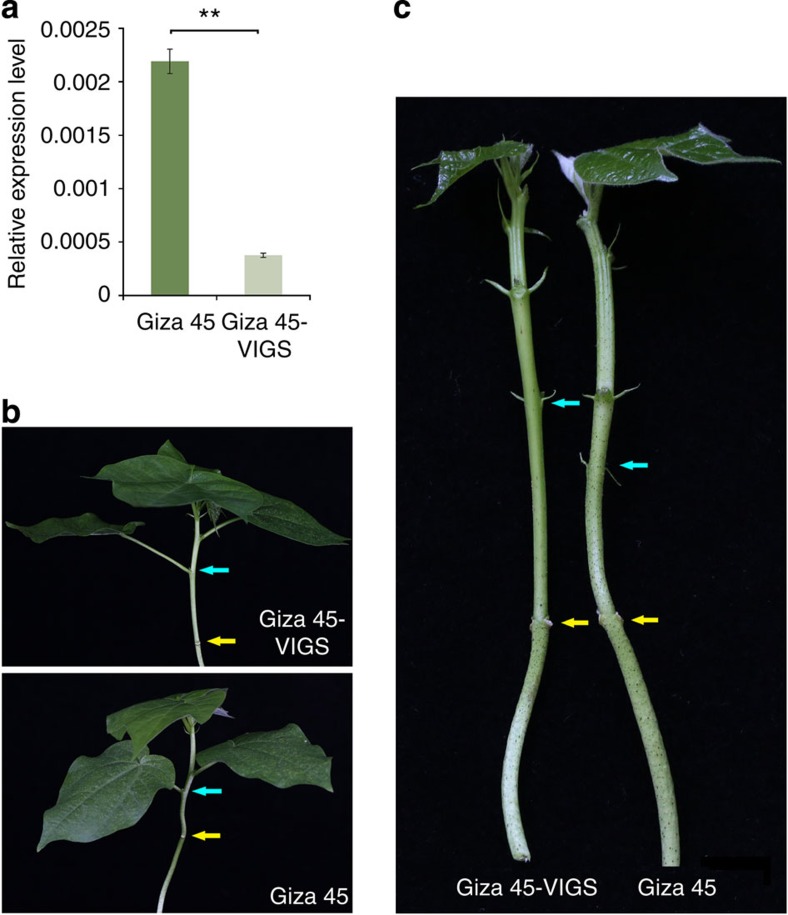
Functional characterization of *GoPGF* by VIGS in *G. barbadense* cv. Giza 45. (**a**) Transcript level of *GoPGF* in normal Giza 45 and corresponding *GoPGF*-silenced leaves. Error bars are s.d. of three biological repeats. **P<0.01; Student's *t*-test, *n*=3. (**b**,**c**) Phenotypes of Giza 45 before and after *GoPGF* silencing by VIGS. Yellow and blue arrows indicate the cotyledonary node and the first vegetative branch, respectively.

To further assess its function, we cloned the 3′-end fragment (904–1,428 bp) of *GbPGF_A12* from Giza 45 and inserted it into pTRV2 for virus-induced gene silencing (VIGS)^25^ to suppress the expression of endogenous *GbPGF* in two cultivated glanded tetraploid cottons. The *PGF*-silenced *G. barbadense* cv. Giza 45 (wild type of the Hai-1 mutant), Hai7124 and *G. hirsutum* acc.TM-1 plants all exhibited a glandless or low gland number phenotype in the newly emerging tissues 14 days post-agro-infiltration (Figs 3a and 4; Supplementary Fig. 6). We observe no or fewer visible glands in the new growing upper stems and leaves of the *PGF*-silenced plants, but the stems below the cotyledon node and cotyledons had many thickly dotted glands that had already formed before infiltration. The transcripts of *GbPGF* in the *PGF*-silenced leaves were significantly reduced compared to the untreated TM-1 and Hai7124, indicating that *GbPGF* was effectively silenced in VIGS plants (Figs 3b and 4a). In the wild-type cotton plants, large cavities of mature pigmented glands were present, however, no such gland cavities were observed in the mesophyll cells of *GbPGF*-silenced leaves in either Hai7124 or TM-1 (Fig. 3c). These data further suggest that *GbPGF* regulates the formation of glands that act as a storage organ for gossypol and other related sesquiterpenes in cotton. Consistently, by suppressing the *GoPGF* expression, gland cell differentiation was blocked, and hemigossypol and gosypol content was reduced by over 93% in the *GbPGF*-silenced leaves compared with the untreated TM-1 and Hai7124 leaves (Fig. 3d), suggesting that *PGF* is also involved in gossypol biosynthesis, directly or indirectly. Interestingly, however, the non-glandular hair trichomes developed normally (Supplementary Fig. 7) in the *PGF*-silenced *G. hirsutum*, suggesting that development of hair or non-grandular and grandular trichomes may use different genetic machinery in cotton.

### Gossypol biosynthesis is not linked to gland formation

Through an antisense strategy, we developed transgenic cotton plants with seed-specific silencing of (+)-δ-cadinene-8-hydroxylase (*CYP706B1*), a P450 monooxygenase in the gossypol biosynthesis pathway^26^. Gossypol levels were significantly reduced in the transgenic seeds, but lysigenous glands still formed as in non-transgenic plants (Supplementary Fig. 8). This result is similar to a previous report, which showed that silencing of (+)-δ-cadinene synthase gene (*CDN*)^27^ that catalyses the first step in gossypol biosynthesis did not affect gland formation, suggesting the terpenoid aldehyde synthesis and gland formation are uncoupled.

**Figure 5:**
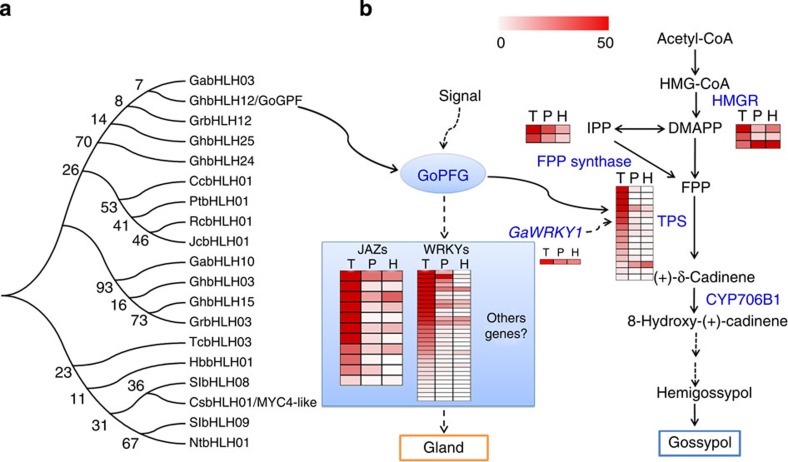
A proposed model of gossypol biosynthesis and gland formation. (**a**) Among 111 bHLH members (Supplementary Data 2), 19 bHLHs from the distinct clade II may be responsible for glandular trichome formation. Numbers on branch nodes indicate percentage support in 1,000 bootstrap trials. (**b**) A proposed model of gossypol biosynthesis and gland formation. Blue text indicates genes downregulated in *GoPGF*-silenced TM-1. Heat map shows gene expression level with FPKM value in VIGS-free TM-1 (AT), *PGF*-silenced TM-1 (P) and Hai-1 (H), each block indicates one gene. First, the expression of *GoPGF* is induced by exogenous signal including JA. Then, GoPGF protein, as a regulator, controls the specification and differentiation of gland cells through regulating the expression of *JAZ*, *WRKYs* or other genes. On the other hand, GoPGF can specifically interact with the G-box motif, which is commonly found in *TPSs* and *WRKYs* promoter regions to feedback on gossypol biosynthesis pathway.

Phylogenetic analysis shows a distinct ‘glandular trichome formation' clade. Together with other bHLH members in the vascular plants species covered by glandular trichome such as *Nicotiana tabacum*, *Solanum lycopersicum* and *Artemisia annua* (Supplementary Fig. 9; Supplementary Data 2), GoPGF is classified in a distinct clade (clade II), which has 19 unknown function members. These bHLHs form a specific cluster involved in regulating glandular trichome formation, distinct from other bHLHs known to be involved in secondary metabolism such as *AtMYC2*, *AtMYC3* and *AtMYC4* in *Arabidopsis*^28^ (Fig. 5a).

### Differential gene expression in *GoPGF*-silenced plants

To gain insight into the regulatory networks that may underlie gland formation, we compared gene expression in leaves of *GoPGF*-silenced plants, the control TM-1 and the mutant Hai-1 by RNA sequencing (RNA-seq). We found that 3,582 genes were deferentially expressed with 2,276 upregulated and 1,306 downregulated in the *GhPGF*-silenced leaves, including significantly reduced expression of 15 terpenoid synthase (*TPS*) genes, 18 *MYBs* and 31 *WRKYs* (Supplementary Data 3). The upregulated genes include genes related to light reaction, cell wall degradation and protein synthesis. Strikingly, the downregulated genes include genes related to secondary metabolism and terpenoid biosynthesis^29^, jasmonate (JA) signalling and genes in the *WRKY* and *MYB* transcription factor families (Supplementary Fig. 10). Subsequent qPCR confirmed decreased expression of some *TPS* and *WRKY* genes in *PGF*-silenced tissues (Supplementary Fig. 11). Yeast one-hybrid binding analysis suggests that *GoPGF* could specifically interact with the G-box motif (Supplementary Fig. 12), which is commonly found in *TPSs* and *WRKYs* promoter regions including *CDN-1* (ref. 29). Pathway analysis also revealed these differentially expressed genes included genes involved in secondary metabolism (Supplementary Table 4). We also found that expression of *GoPGF* was induced by JA treatment (Supplementary Fig. 13). We speculate that GoPGF protein could control the specification and differentiation of gland cells, possibly through regulating the expression of *JAZ*, *WRKYs* or other genes. In addition, *PGF* may also regulate sesquiterpene biosynthesis via binding to the promoters of *TPSs* or *WRKYs* (Fig. 5b).

## Discussion

We have provided evidence that *GoPGF*, encoding a bHLH transcription factor, is likely the causative gene for the glandless phenotype in cotton and appears to be a regulator of glandular trichome formation. It therefore likely represents the first gene to be successfully cloned in tetraploid cotton using a map-based cloning strategy. Its cloning helps to elucidate the molecular mechanism of the genetic control networks involved in secondary substances and formation of glandular trichome, which are storage organs in other plants such as *N. tabacum*^30^, *S. lycopersicum*^31^, *A. annua*^32^ and so on. We identified a bHLH cluster that may have diverged from other bHLH genes and be involved in grandular trichome formation. Glandular trichomes are metabolic hotspots for biosynthesis, regulation and release of numerous volatile and non-volatile phytochemicals used by plants for interacting with the biotic environment. Natural products synthesized in trichomes are also widely adapted as flavorants, perfumes and pharmaceuticals. The distinct glandular trichome formation clade will provide us important candidates to control the biosynthesis of useful secondary compounds through genetic engineering.

Our results further clarified the close and complicated relationship between gland and gossypol. Our study showed glands developed normally in *CYP706B1*-silenced transgenic cotton with reduced gossypol content, suggesting that gossypol was not required for gland morphogenesis, indicating they were uncoupled and controlled by different molecular machines. Nevertheless, gossypol content was markedly suppressed in the *GoPGF*-suppressed cotton without noticeable glands, suggesting that suppression of gland formation will feedback to gossypol biosynthesis possibly through regulating the expression of gossypol-related genes such as *TPSs* and *WRKYs* by binding to their promoters. Taken together, cloning and functional assessment of PGF will open novel opportunities to decipher the mechanism of glandular trichome development in plants.

Australian *Gossypium* species such as *G. australe*, *G. bickii* and *G. sturtianum*, contain immature lysigenous glands but no terpenoid aldehydes, so the pigment glands only appear after seed germination; thus, the dormant seeds of these species lack gossypol^33^,^34^. This distinguishing characteristic, known as the delayed gland morphogenesis trait, has the potential to enable the large scale, direct usage of cottonseed. Various efforts have been made to introduce this unique characteristic of wild Australian cotton species into cultivated tetraploid cotton^35^, but such cultivars with the delayed gland morphogenesis trait have not been developed in cultivated tetraploid cottons by traditional breeding methods. The cloning of *GoPGF* may offer the opportunity to develop such cultivars as we investigate the role of *GoPGF* in the delayed gland formation in Australian species. These discoveries may help further to improve the productivity and economic value of cotton.

## Methods

### Plant materials

A mutant, “Hai-1” (*G. barbadense*), with a glandless trait controlled by a dominant gene, *Gl*_*2*_^*e*^ (refs 17, 19), was gifted by the Cotton Research Institute, Chinese Academy of Agricultural Science (CAAS). N1 and N7 are near isogenic lines of dominant glandless traits in Upland cotton. Three F_2_ mapping populations of 1,599, 244 and 354 individuals, respectively, were developed by crossing TM-1 as a female parent to three glandless lines; Hai-1, N1 and N7. The gland traits of leaves were investigated in 2,197 individuals. TM-1 (*G. hirsutum*), Giza 45 and Hai7124 (*G. barbadense*), which have glands throughout the plants, and three other Upland cotton germplasms, 2(Gl_2_gl_3_) and 2(gl_2_Gl_3_), which have a few glands, and 2(gl_2_gl_3_), which has no glands, were used in this study. All cultivars were planted at Jiangpu Experiment Station and in green houses at Nanjing Agriculture University. Fresh leaves from the cultivars served as the source of genomic DNA, and other vegetative and reproductive tissues were collected for total RNA extraction. RNA was also extracted from developing embryos excised from ovules obtained from each boll at 3, 0, 3, 5, 10 and 20 days postanthesis. All collected plant materials were immediately frozen in liquid nitrogen and stored at −70 °C.

### Map-based cloning of *Gl*_*2*_^*e*^ gene

A previous study reported that the *Gl*_*2*_^*e*^ locus lies between the molecular markers CLR362, NAU2251b, NAU3860b and STV033, with genetic distances of 9.27 and 0.96 cm^21^, respectively. The genetic markers were plotted to the scaffolds of *G. raimondii*^22^. The *Gl*_*2*_^*e*^ locus was mapped to a 1-Mb region flanked by CLR362 and NAU2251b on scaffold D8, namely chr. 12, based on the linkage map constructed by JoinMap 3.0 (ref. 36). By using 598 F_2_ mutant plants with additional molecular markers developed in this work (Supplementary Data 1), the *Gl*_*2*_^*e*^ locus was further mapped to a 43-kb region between SSR marker w7954 and w5383. Then, the genomic DNA sequence of the candidate gene was amplified from *G. arboreum* cv. Jianglinzhongmian (A genome), *G. raimondii* (D genome), TM-1, Giza 36, Giza 45, Giza 67, Giza 80, Junhai-1, Hai7124 and Hai-1 (A_t_D_t_ genome) using primers (Supplementary Data 1), and the PCR products were confirmed by sequencing.

### Subcellular localization of *GoPGF*

To examine the subcellular localization of *GoPGF* in cells, the PCR fragment amplified from cDNA from Hai-1 using the primers K0016F and K0016R (Supplementary Data 1) and inserted into transient expression vector pBinGFP4 (ref. 37) and generated the constructs pBinGFP4::*GoPGF*. The constructs were introduced into *Agrobacterium tumefaciens* strain GV3101 by electroporation. The recombinant plasmid was introduced into tobacco (*Nicotiana benthamiana*) leaf cells by *A. tumefaciens* infiltration^37^. Green fluorescent protein signals in the tobacco epidermal cell were examined and photographed using a ZEISS LSM 710 confocal microscope (Zeiss Microsystems) with a × 20 objective lens (Zeiss) at the specific excitation and emission wavelengths 488 and 495–530 nm.

### Quantitative RT–PCR analysis and pyrosequencing

RNA was extracted from the different tissues (root, stem, leaf and embryos) from plants with and without glands using a BioFlux kit. First-strand cDNA was generated using TransScript One-Step gDNA Removal and cDNA Synthesis SuperMix (TransGen Biotec Co., Ltd.) according to the manufacturer's instructions. Quantitative RT–PCR and pyrosequencing were performed with the primers listed in Supplementary Data 1. The pyrosequencing reaction was performed using a PyroMark sequencer (Qiagen, Valencia, CA).

### Promoter activity analysis

The *GoPGF* promoter was isolated with the designed primers H3996F and H3996R from the A subgenome of TM-1 and Hai-1 (Supplementary Data 1). The promoters cover 1,818 and 1,809 bp in length upstream of the transcription start site. The binary vector pBI121 was used as a basic expression vector to make constructs. All the constructs were introduced into *A. tumefaciens* strain GV3101. Individual *Agrobacterium* colonies were grown on Luria-Bertani (LB) plates with kanamycin (50 μg ml^−1^) for 48 h at 28 °C. A single positive colony was used to inoculate a 5-ml culture (LB with 50 μg ml^−1^ kanamycin). Bacteria were pelleted, resuspended in infiltration medium (10 mM MgCl_2_, 10 mM MES and 150 μM acetosyringone (pH 5.6)) to an OD_600_ of 0.5–0.6, then incubated at room temperature for 3 h. The bacterial suspension was infiltrated into the abaxial side of fully expanded 6-week-old *N. benthamiana* leaves using a needleless 1-ml syringe. For each experiment, the positive control (pBI121 intron with 35S-GUS), the negative control (TATA-GUS in pMDC162 intron), and the constructs under investigation were infiltrated in areas of the same leaf or different leaves. After infiltration, the plants were kept in the greenhouse for 72 h for inoculation. For histochemical staining, the plant tissues were incubated at 37 °C overnight (12 h) in the dark in 1 mM X-Gluc (5-bromo-4-chloro-3-indolyl-b-D-glucuronide) in 100 mM sodium phosphate (pH 7.0), 10 mM EDTA, 0.5 mM potassium ferricyanide, 0.5 mM potassium ferrocyanide, 0.3% (v/v) Triton X-100 and 20% (v/v) methanol to eliminate endogenous GUS activity. After 12 h of staining, tissues were destained in an ethanol series (50, 70 and 95%) to remove chlorophyll, stored in 70% (v/v) ethanol, and photographed with a digital camera^38^.

### Virus-induced gene silencing assay

To knockdown the expression of *PGF* gene, a 415-bp fragment of *GbPGF_A12* cDNA from TM-1 corresponding to bases 904–1,319 of the *PGF* gene was PCR-amplified using Pfu DNA polymerase (Promega) and primers H1238F and H1238R (Supplementary Data 1). The resulting PCR product was cloned into *Xba*I-*BamH* I-cut pTRV2 (ref. 25) to produce a VIGS vector named pTRV2-*PGF_A12*. The vectors pTRV1 and pTRV2-*PGF_A12* were introduced into the *Agrobacterium* strain GV3101 by electroporation (Bio-Rad, Hercules, CA, USA). For the VIGS assay, the transformed *Agrobacterium* colonies containing pTRV1 and pTRV2-*PGF_A12* were grown overnight at 28 °C in an antibiotic selection medium containing rifampicin and kanamycin 50 mg l^−1^ each. *Agrobacterium* cells were collected and resuspended in infiltration medium (10 mM MgCl_2_, 10 mM MES and 200 μM acetosyringone), adjusted to an OD_600_ 0.5. *Agrobacterium* strains containing TRV1 and TRV2 vectors were mixed at a ratio of 1:1. Seedlings with mature cotyledons but without a visible rosette leaf (7 days after germination) were infiltrated by inserting the *Agrobacterium* suspension into the cotyledons via a syringe. The plants were grown in pots at 25 °C in a growth chamber under a 16-h light per 8-h dark cycle with 60% humidity.

### Histochemistry and microscopy

Leaves detached from the seedlings before and after the treatment were cut into 1 mm^2^ pieces and fixed in 0.1 mol l^−1^ phosphate buffer (pH 7.0) containing 2.5% glutaraldehyde at 4 °C overnight. After three 30-min rinses in 0.1 mol l^−1^ phosphate buffer (pH 7.0), samples were postfixed in 0.5% osmium tetroxide solution in buffer at 4 °C for 3 h. The samples were then rinsed in 0.1 mol l^−1^ phosphate buffer (pH 7.0) for 30 min three times. Samples were dehydrated through a series of ethanol solutions (30, 40, 50, 65, 80, 90 and 100%; 30 min in each) twice, with a final change to 1, 2-epoxypropane, and were then embedded in Epon 812. Semi-thin sections (1–2 μm) were cut using a Reichert-Jung ultramicrotome and stained with toluidine blue O or methylene blue. Sections were examined and digitally recorded on a microscope.

### Gossypol detection and analysis

The total gossypol concentration in leaves from TM-1, *PGF-*silenced TM-1, Hai-1, 2(Gl_2_gl_3_), 2(gl_2_Gl_3_) and 2(gl_2_gl_3_) plants was determined by high-performance liquid chromatography (HPLC)^39^. Each 100 mg of freeze-dried plant sample was dissolved with 1 ml leaf extraction (acetonitrile/water/phosphoric acid=80:20:0.1) for 1 h. The leaf extraction was centrifuged at low speed for 5 min and then transferred the supernatant carefully at room temperature. The eluent was filtered through a 0.22-μm nylon filter into a vial for HPLC analysis with Agilent Zorbax eclipse XDB-C18 analytical column (150 × 4.6 mm, 5 micron). The column was eluted with buffer (EtOH/MeOH/IPA/ACN/H_2_O/EtOAc/DMF/PPAcD=16.7:4.6:12.1:20.2:37.4:3.8:5.1:0.1) and kept at 35±1 °C during the procedure. The determination wave length was 272 nm. Standards of gossypol, hemigossypolone, were used to assess the retention time of the individual terpenoids. The concentration of these compounds was calculated using Agilent 1100 system by comparing to the gossypol standard curve. All the reagents were of analytical grade and were made in China, with the exception of gossypol, which was purchased from Sigma Chemical Co.

### RNA-seq

Total RNA samples were quality-checked using RNA Pico Chips on an Agilent 2100 bioanalyzer. All RNA samples were quantified and qualified with an RNA integrity number >8. RNA-seq libraries were constructed following the Illumina kit recommendations. The constructed libraries, indexed with six nucleotide sequences (barcode), were pooled together with equimolar amounts (2 nM) and were sequenced on the Illumina HiSeq 2000 sequencer with 2 × 100 bp. Raw data have been deposited in GenBank under the accession PRJNA265955. The raw FASTQ format data generated from CASAVA v1.8.2 were first assessed for quality using FASTQC v0.10.1 (http://www.bioinformatics.babraham.ac.uk/projects/fastqc/) and FASTX toolkit v0.0.13.2 (http://hannonlab.cshl.edu/fastx_toolkit/). Poor quality reads (Phred score<20) were trimmed at both ends with SolexaQA packages v2.2 (http://solexaqa.sourceforge.net/); only the reads with lengths ≥25 bp on both sides of the paired-end format were subjected to further analysis. The data were then aligned with the *G. hirsutum* (TM-1) genome (PRJNA248163). The software Cufflinks v2.2.1 (http://cufflinks.cbcb.umd.edu/) was used to accurately quantify the abundance of genes and calculate the fragments per kilobase of genes per million mapped reads (FPKM). Differential expression was defined as a gene with a minimum of a twofold change (TM-1 versus PGF-silenced TM-1 and TM-1 versus Hai-1) with RPKM>1 in either TM-1 or *PGF*-silenced TM-1 or Hai-1. K means clustering was performed with the open-source program, Cluster3.0 (http://bonsai.hgc.jp/~mdehoon/software/cluster/software.htm). The genes in each cluster were then classified into MapMan functional categories^40^. Changes in the significance of expression were investigated in functional categories of the MapMan annotation through the application of a two-sided Wilcoxon rank test with a Benjamini-Yekutieli correction for multiple tests. Pathway analysis was mainly based on the Kyoto Encyclopedia of Genes and Genomes (KEGG) database^41^.

### Development of the *CYP706B1* RNAi transgenic line

To unravel the relationship between gossypol and glands, we cultivated the *CYP706B1* RNAi transgenic lines. A 408 bp fragment of *CYP706B1* (GenBank: AF332974.1) was amplified from the leaf cDNA library with dsP1-F-*Bam*HI: 5′-GGATCCTCAGCTCGTATTCATGGCTG-3′ and dsP1-R-*Xba*I: 5′-TCTAGACAAATACAATATCATTGAGG-3′ primers, and dsP1-F-*Sac*I: 5′-GAGCTCTCAGCTCGTATTCATGGCTG-3′ and dsP1-R-*Not*I: 5′-GCGGCCGCCAAATACAATATCATTGAGG-3′ primers. The dsRNA construct was inserted into the *Bam*HI-*Sac*I site of binary vector pCAMBIA2301. The 1,143 bp globulin promoter, Proglobulin, was amplified with PG-F-*Hin*dIII: 5′-AAGCTTCTATTTTCATCCTATTTAGA-3′ and PG-R-*Bam*HI: 5′-GGATCCGATTACGATAAGCTCTGTAT-3′ primers from cotton genomic DNA and inserted to the *Hin*dIII/*Bam*HI site to control dsRNA construct expression. The resultant constructs of Proglobulin::dsCYP706B1 (P1) was transferred into *A. tumefaciens* strain LBA4404 and then used for cotton transformation. Cotton seeds (R15) were surface-sterilized with 70% ethanol for 1 min, and 15% H_2_O_2_ for 4 h, followed by washing with sterile water five times. The sterilized seeds were germinated on Murashige and Skoog (MS) medium under dark conditions at 28 °C for 7 days. Hypocotyls were cut into 1-cm fragments and incubated with the overnight culture of *Agrobacterium* for 20 min. After 2 days of co-cultivation, hypocotyl explants were transferred to MS medium containing 1 μg ml^−1^ 2,4-Dichlorophenoxyacetic acid (2,4-D), 50 μg ml^−1^ kanamycin and 400 μg ml^−1^ cefalotin for 2 months. The resistant calli were transferred to hormone- and antibiotic-free MS medium for somatic embryogenesis and plant regeneration. The resultant transgenic plants were transferred into soil for growing. Seeds from transgenic line P1-13-8 and progeny plants showed low gossypol contents. Genomic DNA from P1-13-8 and R15 were prepared for Southern blot analysis. *Hin*dIII- or *Eco*RI-digested genomic DNAs were separated on a 1% agarose gel, transferred to a Hybond-N^+^ nylon membrane (GE Healthcare Life Sciences) and probed with *NPTII* gene. The DIG High Primer DNA Labeling and Detection Starter kit II (Roche Applied Science, USA) were used for labelling and hybridization according to the manufacturer's protocol.

### Yeast one-hybrid assay

The yeast one-hybrid assay was performed using the MATCHMAKER one-hybrid system (Clontech). Fragments containing four tandem copies of G-box (5′-CACGTG-3′) (4 × G-box WT) and G-box mutant (5′-CATAGA-3′; 4 × G-box mutant) were synthesized by GenScript Biotechnology Co., Ltd. These two fragments were ligated into the *Hin*dIII-*Xho*I sites of pAbAi. The bait constructs were linearized with *Bst*BI and integrated into the yeast genome (strain Y1H). Various concentrations of aureobasidin A (AbA; Clontech, cat. no. 630446) on SD-Ura medium were used to identify the basal expression of AUR1-C. The ORF of *GoGPF* was ligated to the GAL4 activation domain in pGAD424. Yeast transformants were tested on SD/-Ura medium containing 20 ng ml^−1^ AbA.

### Phylogenetic analysis

Alignment of the amino acid sequences of the *GoPGF* and bHLH families in other species was performed using the CLUSTALX program^42^. A neighbour-joining (NJ) method was then applied to produce a phylogenetic tree. The relative degree of branch support was determined within the NJ framework using the bootstrap procedure. The original data set was resampled 1,000 times.

## Additional information

**Accession codes:** Sequences have been deposited at DDBJ/EMBL/GenBank under the accessions KP072743 (*GoPGF_A12* of TM-1), KP072744 (*GoPGF_D12* of TM-1), KP072745 (*GoPGF_A12* of Hai-1) and KP072746 (*GoPGF_D12* of Hai-1). Transcriptome data have been deposited in GenBank under the accession PRJNA265955.

**How to cite this article:** Ma, D. *et al*. Genetic basis for glandular trichome formation in cotton. *Nat. Commun.* 7:10456 doi: 10.1038/ncomms10456 (2016).

## Supplementary Material

Supplementary Information Supplementary Figures 1-13 and Supplementary Tables 1-4

Supplementary Data 1 All primers used in this study

Supplementary Data 2 List of bHLH genes form different species

Supplementary Data 3 Gene list of transcripts with decreased and increased expression in the leaves after GoPGF silenced by VIGS
