# Supplementary material for: Genetic basis for glandular trichome formation in cotton
Source: Nat Commun. 2016 Jan 22;7:10456. doi: 10.1038/ncomms10456 (PMC4735898; doi:10.1038/ncomms10456)
Supplement: Supplementary Information — Supplementary Figures 1-13 and Supplementary Tables 1-4 [file ncomms10456-s1.pdf]

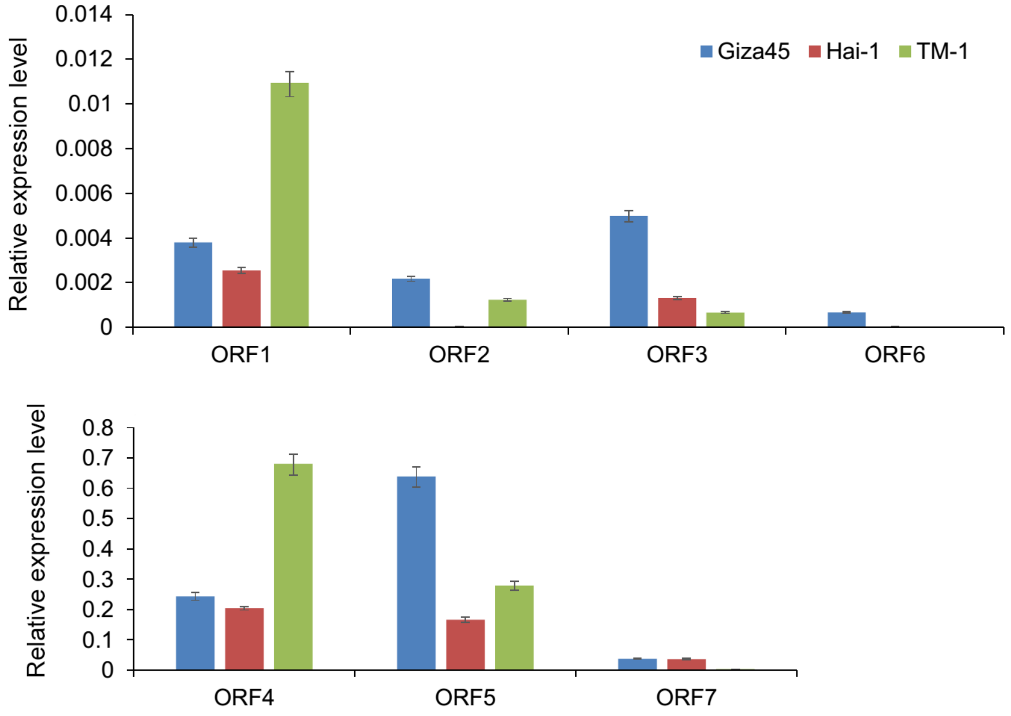

Supplementary Figure 1: Comparison of the expression level of the seven candidate ORFs in glanded and glandless cotton species.

Expression analysis of seven candidate genes in leaves of Giza 45, Hai-1 and TM-1. The error bar represents the standard deviation of the mean values of three biological replicates. The histone 3 gene is used as the internal control.

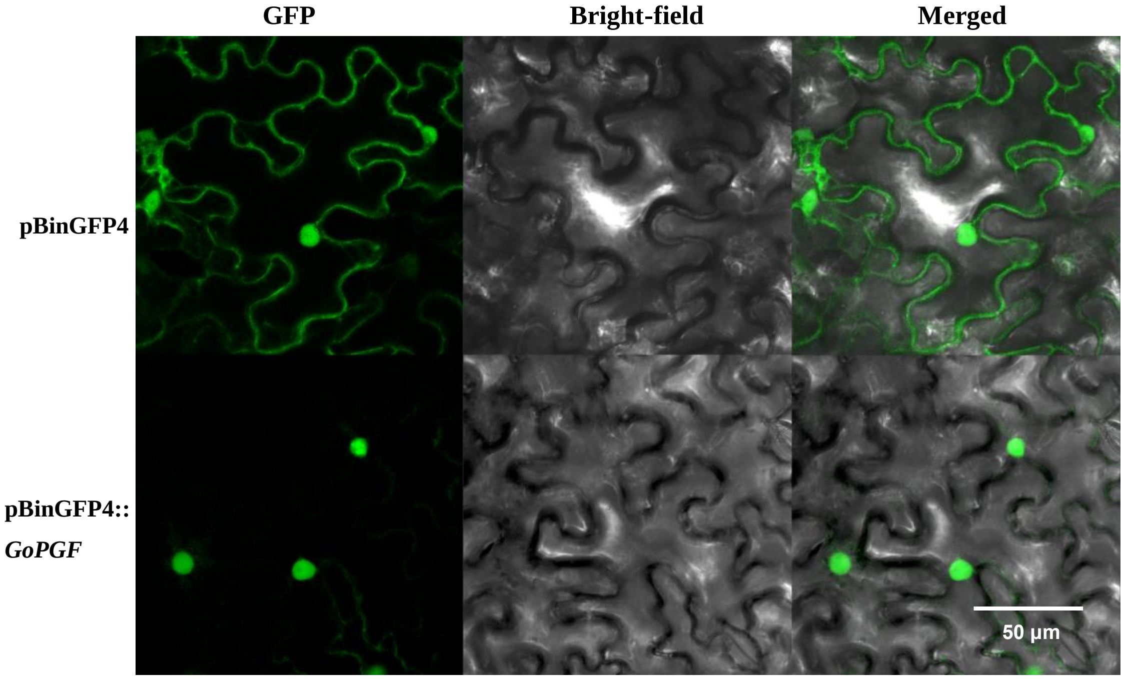

Supplementary Figure 2: Subcellular localization of GoPGF-GFP.

GoPGF-GFP is transiently expressed in tobacco leaf cells. pBinGFP4 construct is used as the control. Mergers display the overlay of bright field and green fluorescence images.

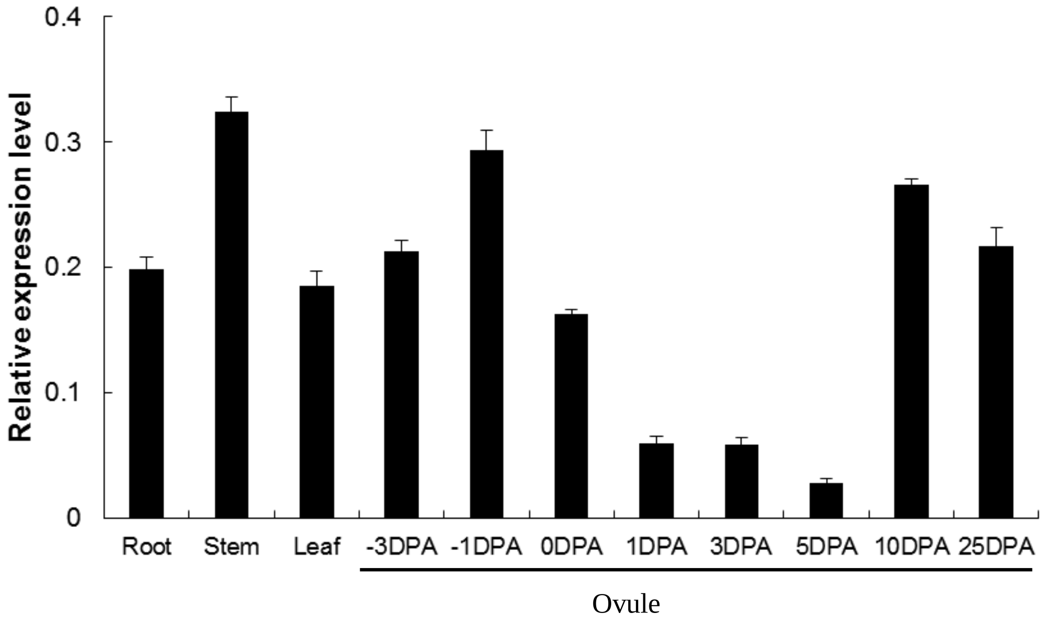

Supplementary Figure 3: Expression pattern of *GoPGF* gene in different tissues and ovule developmental stages of cotton.

qRT-PCR expression analysis of *GoPGF* in root, stem, leaf and ovules at -3, -1, 0, 3, 5, 10, 25 day post-anthesis (DPA) of *G. hirsutum* acc. TM-1. The error bar represents the standard deviation of the mean values of three biological replicates. The histone 3 gene is used as the internal control.

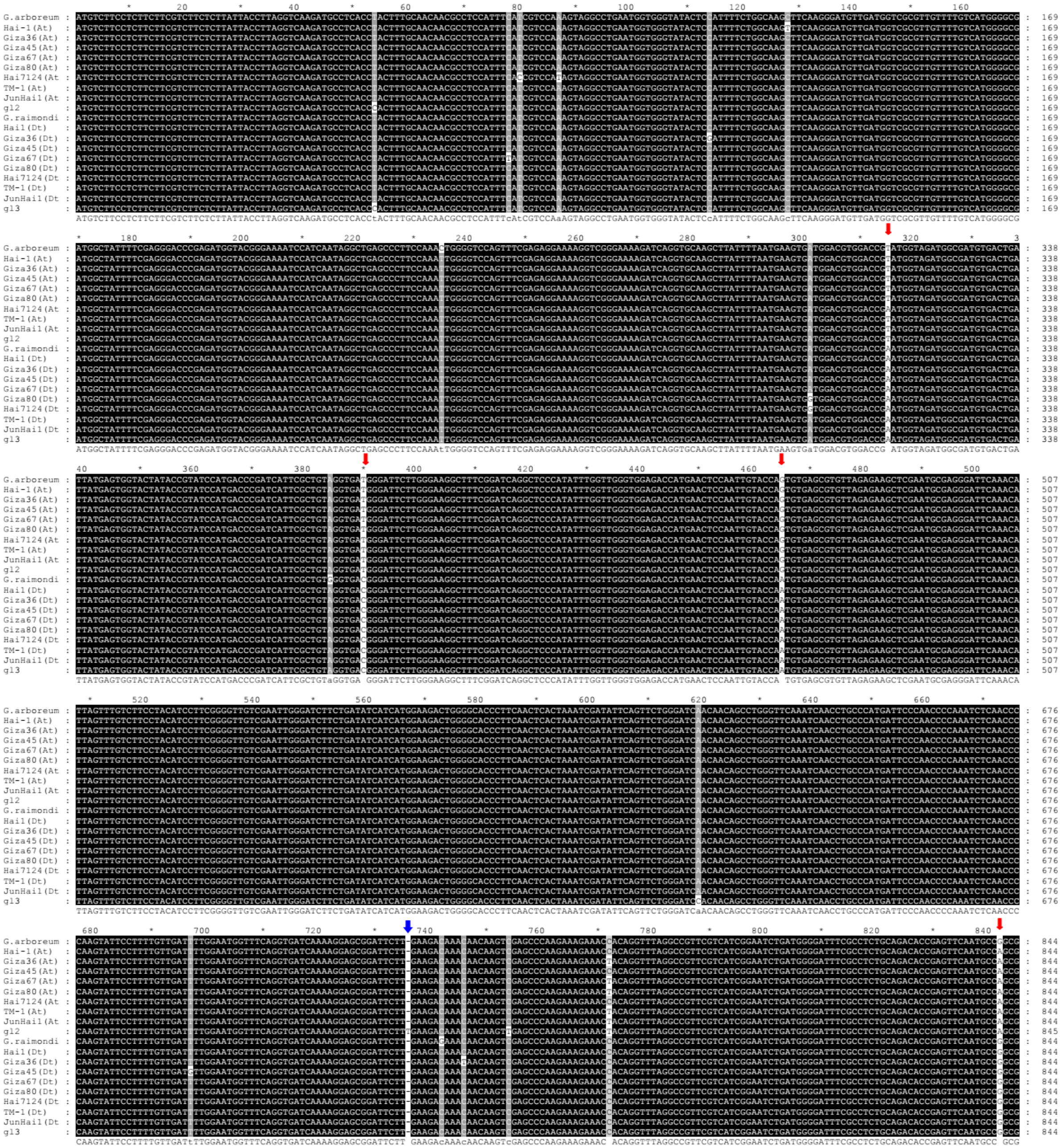

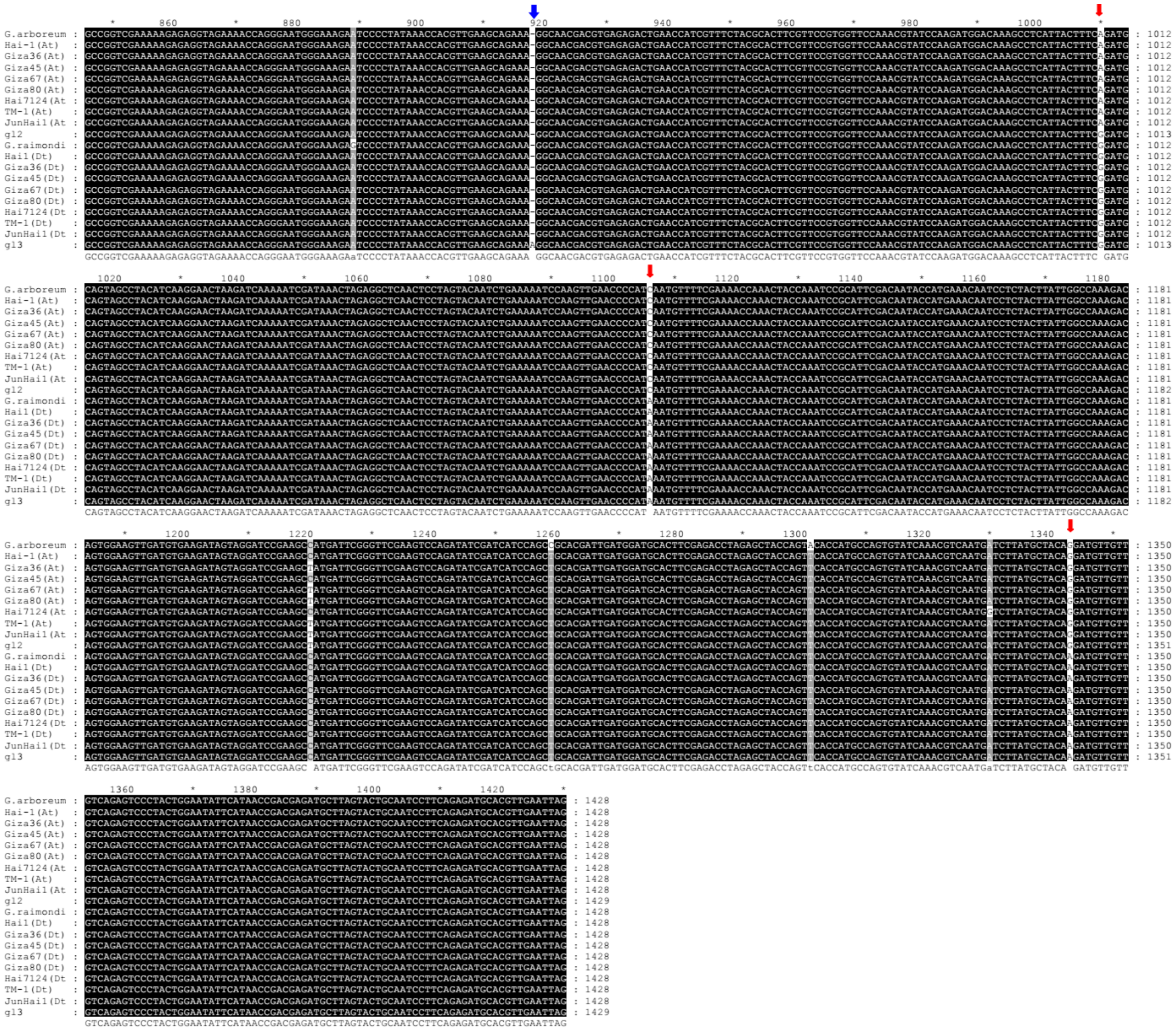

Supplementary Figure 4: Alignment of the gene sequences of the *GoPGF_A12* and *GoPGF_D12* from the varieties used in this study. Red arrows mark seven SNPs between homoeologous gene pairs *GoPGF_A12* and *GoPGF_D12* (Detailed information about these SNP are list in Supplementary Table 3). *GhPGF_A12*^{m} (gl_2) and *GhPGF_D12*^{m} (gl_3) are two recessive mutant genes corresponding to *GhPGF_A12 and GhPGF_D12*. Blue arrows indicate “T” nucleotide insertion between 735 and 736bp in *GhPGF_A12*^{m} (gl_2) and “A” nucleotide insertion between 916 and 917 bp in *GhPGF_D12*^{m} (gl_3), which create a premature termination codon and result in loss-function of *GoPGF*.

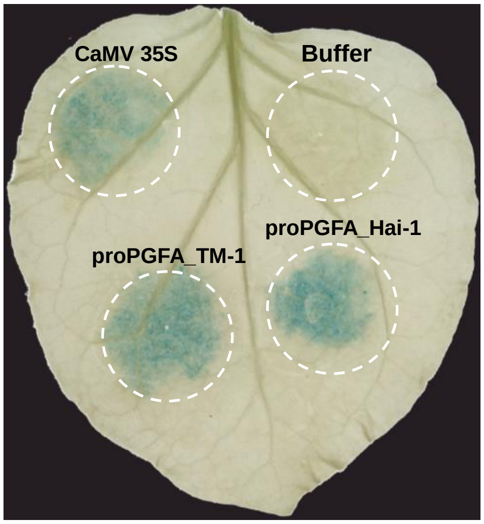

Supplementary Figure 5: Promoters activity assay by histochemical GUS staining in *Nicotiana* transiently expressing leaves. GUS reporter gene is fused to *PGF_A12* from TM-1 (proPGFA_TM-1), Hai-1 (proPGFA_Hai-1) and CaMV35S promoters.

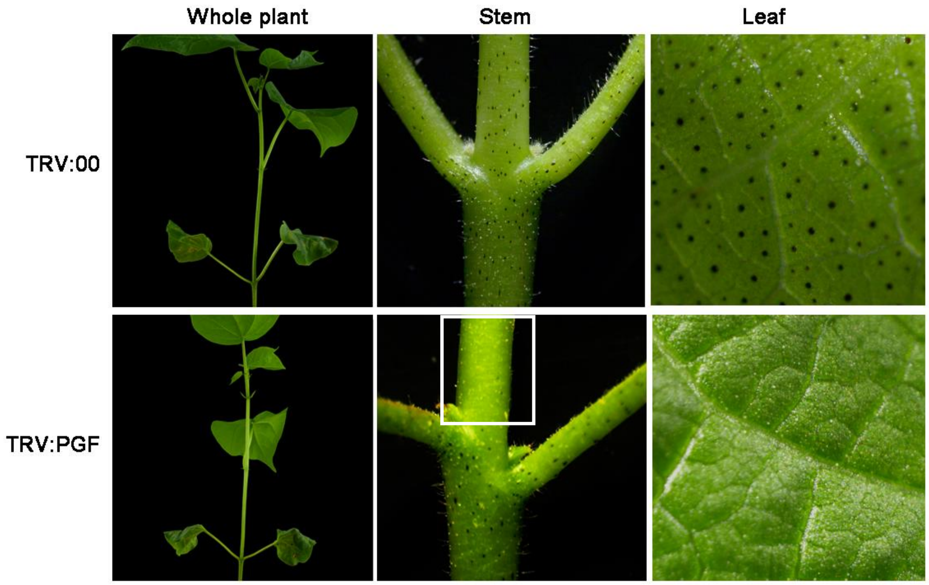

Supplementary Figure 6: Phenotypic change of glands in the stem and leaf of Hai7124 after the expression of *GbPGF* gene is suppressed by VIGS.

Normal gland trait in Hai7124 (up), and glandless trait in the stem and leaf of *GbPGF*-silenced plants by VIGS (down). White box marks the glandless phenotype in the newly emerging stem of the *GbPGF*-silenced plants.

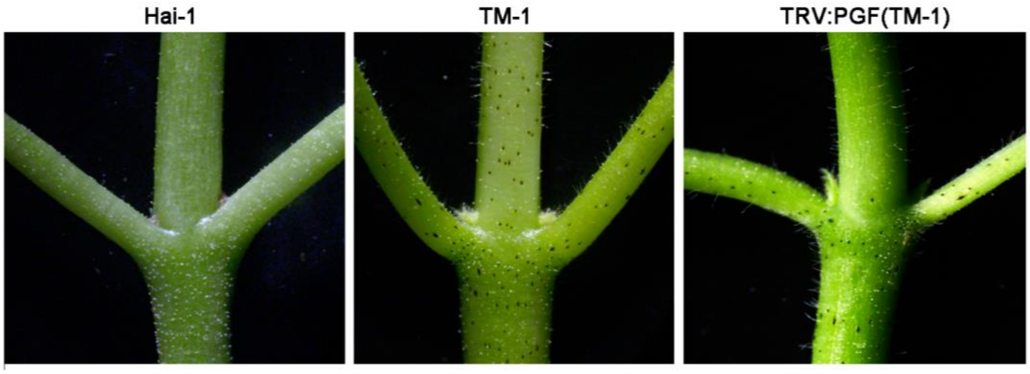

Supplementary Figure 7: Glands and Trichomes on the stems of Hai-1, TM-1 and *PGF*-silenced TM-1 cotton plants.

No glands and stem trichomes (non-glandular) are observed in the stem of Hai-1 (left), and many thickly dotted glands and trochomes covered the stem of TM-1 (middle). After *GoPGF* gene is silenced by VIGS in TM-1, less visible glands are observed on the new born stem, whereas the stem trichomes develop normal (right).

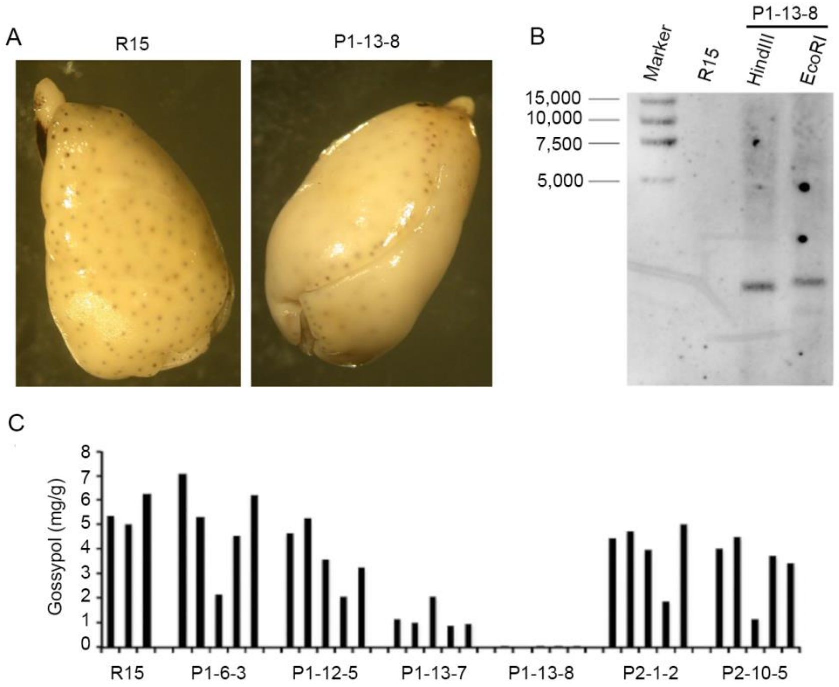

Supplementary Figure 8. Low-gossypol trait in seeds of *CYP706B1* RNAi transgenic cotton plants.

(a) Seed (cotyledon) of the wild-type R15 and *CYP706B1* RNAi transgenic P1-13-8 cotton. Note that although gossypol in P1-13-8 seed is reduced to undetectable level, glands is still visible. (**b)** Southern blot analysis of P1-13-8 transgenic cotton plant. *Hin*d III or *Eco*R I digest genomic DNA of P1-13-8 and *Hin*d III digested genomic DNA of R15 are separated on 1% agarose gel and transferred to Hybond N^+ nylon membrane, and probed with Dig labeled *NPTII* gene fragment. (**c)** Gossypol contents in single seeds of R15 (glanded cotton of *G. hirsutum*) and selected segregates of P1 and P2 constructs by HPLC analysis. Plant P1-13-8 produced seeds with ultra-low levels of gossypol.

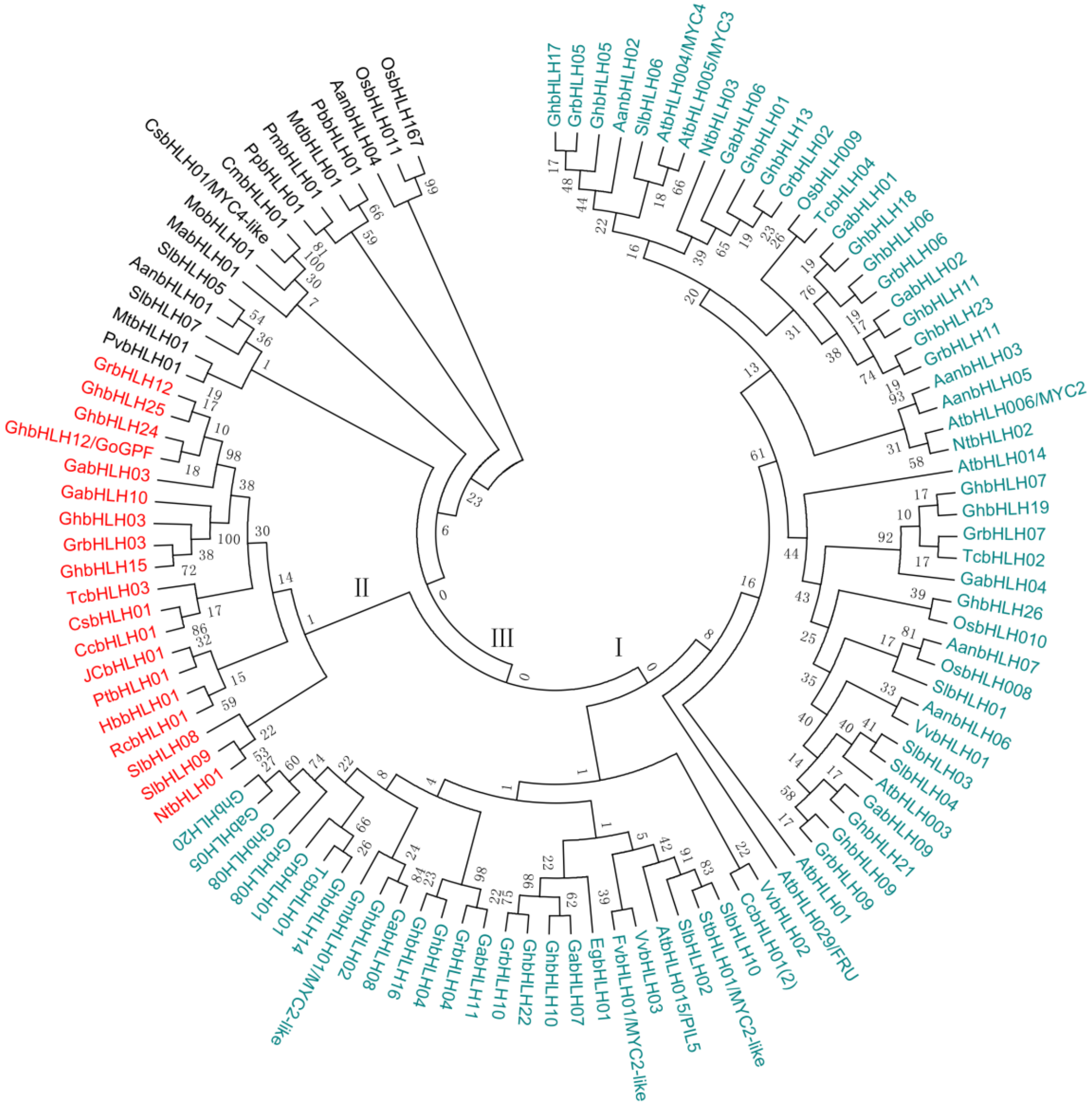

Supplementary Figure 9: Phylogenetic tree of GoPGF and other 110 bHLH members from different species.

A phylogenic tree of GoPGF-clade bHLH proteins based on the alignment of their full-length coding sequences. bHLH genes in the phylogenetic tree are list in Supplementary Data 2. These bHLH genes classified into three main branches (I, II and III). The phylogenetic tree is generated using MEGA 5.0 software. Numbers above branches indicate percent support for the nodes defining the families in distance bootstrap analysis (1000 replicates). Red text indicates a distinct clade (clade II) which likely responsible for glandular trichome formation.

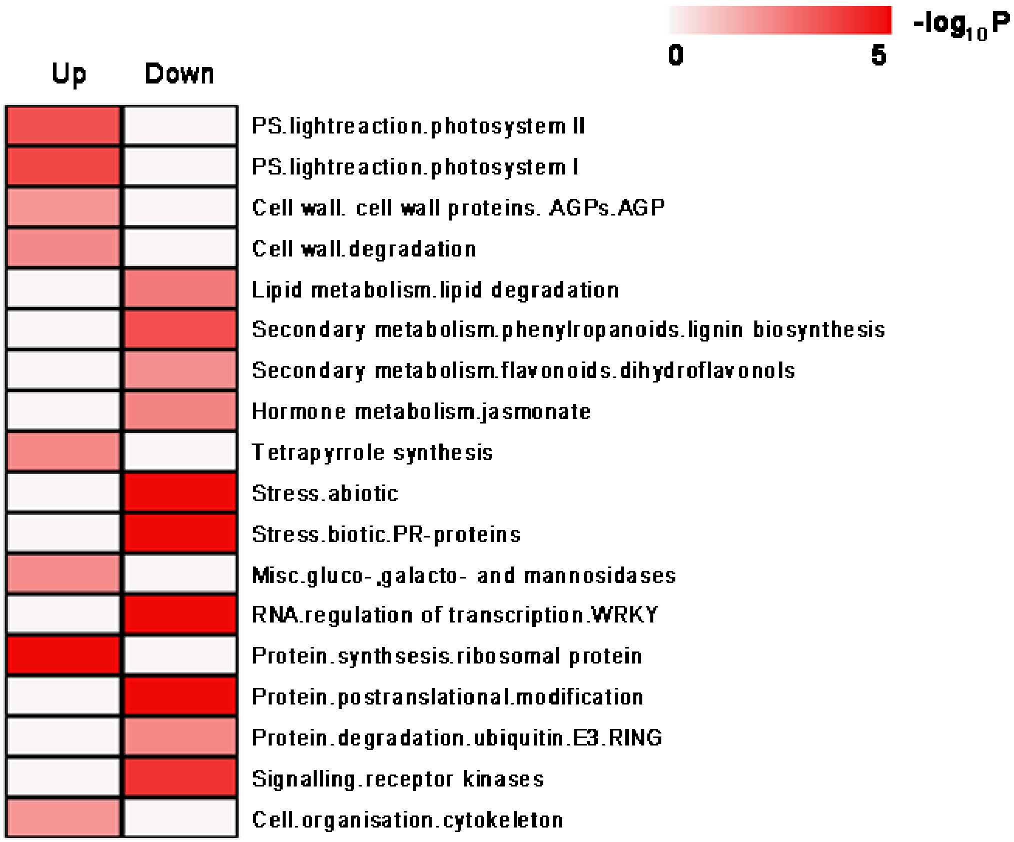

Supplementary Figure 10: Functional enrichment of the transcripts with decreased and increased expressions in leaves after *GoPGF* silenced by VIGS.

Red color indicates that the process is enriched more significantly. The data is subjected to a Bin-wise Wilcoxon test using MapMan software. The resulting *p*-values are adjusted according to Benjamini and Hochberg.

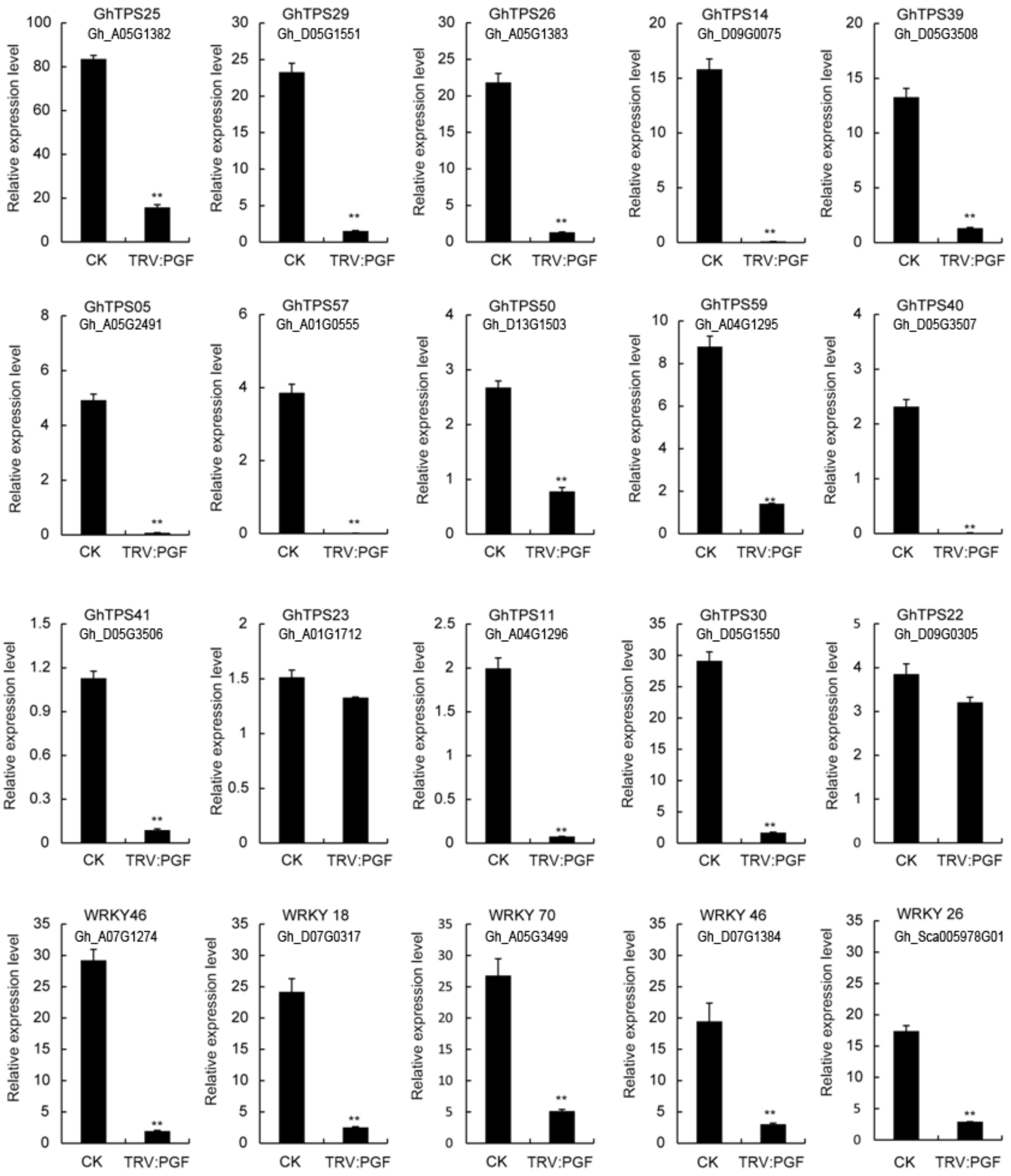

Supplementary Figure 11: qRT-PCR validation of the expression level of *TPS* and *WRKY* genes in *PGF*-silenced leaves of TM-1.

The error bar represents the standard deviation of the mean values of three biological replicates. ** = $p<0.01$, student's t-test, n=3. The histone 3 gene was used as the internal control.

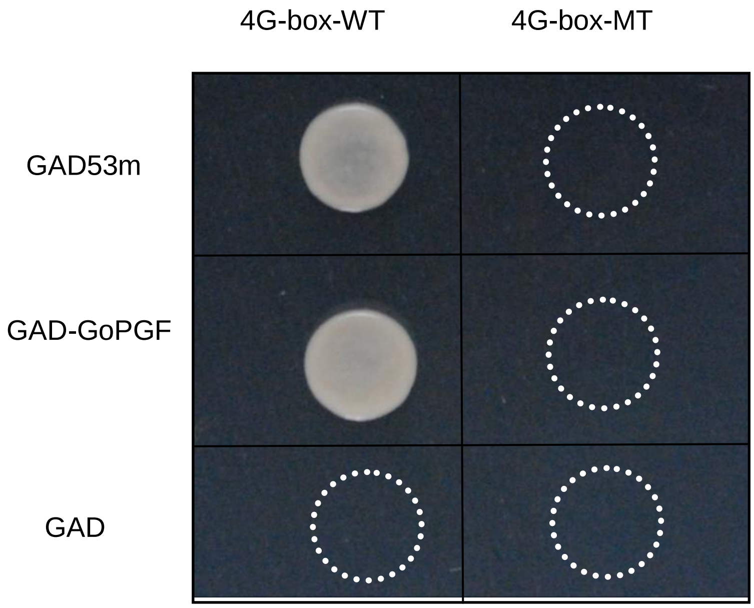

Supplementary Figure 12. GoPGF protein Interacts with G-box in yeast one hybridization assay.

pGAD was used as a negative control, and GAD53m as the positive control. Transactivation analysis of corresponding constructs by yeast one-hybrid was detected on the SD/Trp- and SD/Trp-/His-/20 ng/ml AbA media. 4G-box-WT is a synthesized fragament containg four tandem copies of G-box (5'-CACGTG-3') and 4G-box-MT contains G-box mutant (5'-CATAGA-3').

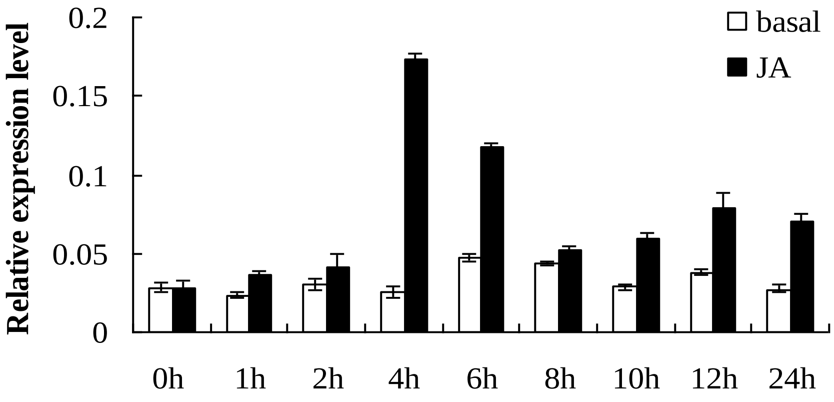

Genes are list in the Supplementary Data 3.

Supplementary Figure 13: Expression time course of *GoPGF* in response to JA treatment by qRT-PCR.

The leaves of TM-1 seedlings are treated with 100μM MeJA. The error bar represents the standard deviation of the mean values of three biological replicates. The histone 3 gene is used as the internal control.

Supplementary Table 1. Segregation rations for presence and absence of gland

Mapping populations	Total	No. glandless plants	No. glanded plants	Chi-square test	Probability
(N1×TM-1)F_2	244	179	65	0.048 (3:1)	0.25-0.50
(N7×TM-1)F_2	354	257	97	0.105 (3:1)	0.25-0.50
(Hai-1×TM-1)F_2	1599	1188	411	0.010 (3:1)	0.25-0.50
Total	2197	1624	573	0.024 (3:1)	0.25-0.50

χ^2 test for goodness-of-fit at 0.05 significance level

($\chi^2_{0.05:1}$=3.84)

Supplementary Table 2. Seven candidate genes and its putative function

Candidate ORFs	Length (bp)	Putative function
ORF1	978	homeobox 7
ORF2	1428	Basic helix-loop-helix (bHLH) DNA-binding family protein
ORF3	1425	alpha/beta-Hydrolases superfamily protein
ORF4	1125	GDSL-like Lipase/Acylhydrolase superfamily protein
ORF5	1311	Lung seven transmembrane receptor family protein
ORF6	966	branchless trichome
ORF7	1371	growth-regulating factor 8

Supplementary Table 3. SNPs between genomic sequences of *GoPGF_A12* and *GoPGF_D12* in the tetraploid cotton.

**Locus (bp)**	**SNP（A/D）**
315	T/A
390	T/C
465	G/A
841	G/A
1008	A/G
1104	C/A
1341	G/A

Supplementary Table 4. Pathway analysis of the differential expressed genes (DEGs) with decreased and increased expression in the leaves after *GoPGF* silenced by VIGS.

Pathway	DEGs with pathway annotation (911)	all genes with pathway annotation (12774)
Starch and sucrose metabolism	91	727
Phenylpropanoid biosynthesis	33	297
Amino sugar and nucleotide sugar metabolism	32	265
Pentose and glucuronate interconversions	27	284
Phenylalanine metabolism	26	326
Galactose metabolism	21	203
Purine metabolism	20	527
Flavonoid biosynthesis	19	162
Glycolysis / Gluconeogenesis	19	242
Porphyrin and chlorophyll metabolism	17	107
Glutathione metabolism	16	154
Ascorbate and aldarate metabolism	16	161
Carbon fixation in photosynthetic organisms	15	173
Pyruvate metabolism	14	226
Fructose and mannose metabolism	13	122
Phosphatidylinositol signaling system	13	171
Metabolism of xenobiotics by cytochrome P450	13	200
Drug metabolism - cytochrome P450	13	226
T cell receptor signaling pathway	13	249
Pentose phosphate pathway	12	129
Inositol phosphate metabolism	12	138
Tryptophan metabolism	12	217
Glycerophospholipid metabolism	12	231
Fatty acid degradation	12	235
Glycerolipid metabolism	12	243
Retinol metabolism	11	157
Glyoxylate and dicarboxylate metabolism	11	175
Starch and sucrose metabolism	91	727
